# Suction assisted liposuction does not impair the regenerative potential of adipose derived stem cells

**DOI:** 10.1186/s12967-016-0881-1

**Published:** 2016-05-06

**Authors:** Dominik Duscher, Anna Luan, Robert C. Rennert, David Atashroo, Zeshaan N. Maan, Elizabeth A. Brett, Alexander J. Whittam, Natalie Ho, Michelle Lin, Michael S. Hu, Graham G. Walmsley, Raphael Wenny, Manfred Schmidt, Arndt F. Schilling, Hans-Günther Machens, Georg M. Huemer, Derrick C. Wan, Michael T. Longaker, Geoffrey C. Gurtner

**Affiliations:** Hagey Laboratory for Pediatric Regenerative Medicine, Division of Plastic Surgery, Department of Surgery, Stanford University School of Medicine, Stanford, CA USA; Section of Plastic, Aesthetic and Reconstructive Surgery, Johannes Kepler University, Linz, Austria; Institute for Stem Cell Biology and Regenerative Medicine, Stanford University School of Medicine, Stanford, CA USA; Department of Plastic Surgery and Hand Surgery, Technical University Munich, Munich, Germany

## Abstract

**Background:**

Adipose-derived stem cells (ASCs) have been identified as a population of multipotent cells with promising applications in tissue engineering and regenerative medicine. ASCs are abundant in fat tissue, which can be safely harvested through the minimally invasive procedure of liposuction. However, there exist a variety of different harvesting methods, with unclear impact on ASC regenerative potential. The aim of this study was thus to compare the functionality of ASCs derived from the common technique of suction-assisted lipoaspiration (SAL) versus resection.

**Methods:**

Human adipose tissue was obtained from paired abdominoplasty and SAL samples from three female donors, and was processed to isolate the stromal vascular fraction. Fluorescence-activated cell sorting was used to determine ASC yield, and cell viability was assayed. Adipogenic and osteogenic differentiation capacity were assessed in vitro using phenotypic staining and quantification of gene expression. Finally, ASCs were applied in an in vivo model of tissue repair to evaluate their regenerative potential.

**Results:**

SAL specimens provided significantly fewer ASCs when compared to excised fat tissue, however, with equivalent viability. SAL-derived ASCs demonstrated greater expression of the adipogenic markers FABP-4 and LPL, although this did not result in a difference in adipogenic differentiation. There were no differences detected in osteogenic differentiation capacity as measured by alkaline phosphatase, mineralization or osteogenic gene expression. Both SAL- and resection-derived ASCs enhanced significantly cutaneous healing and vascularization in vivo, with no significant difference between the two groups.

**Conclusion:**

SAL provides viable ASCs with full capacity for multi-lineage differentiation and tissue regeneration, and is an effective method of obtaining ASCs for cell-based therapies.

## Background

Adipose tissue has recently been identified as a promising source of multipotent cells for use in regenerative medicine. Adipose-derived stem cells (ASCs) are cells of mesenchymal origin with a capacity to differentiate through adipogenic, osteogenic, and chrondrogenic lineages, among others [1, 2]. Notably, in contrast to bone marrow-derived mesenchymal stem cells (BM-MSCs), ASCs derived from adipose tissue are abundant [3] and relatively easily obtainable [1, 2, 4]. Due to their high yield in adipose tissue, ASCs additionally have the potential to be used in clinical therapy without the need for expansion in culture.

The potential utility of ASCs in tissue engineering and cell-based regenerative therapies has been confirmed in a variety of pre-clinical and clinical applications. For example, pullulan-collagen hydrogel scaffolds seeded with ASCs have been demonstrated to increase vascularity and improve wound healing [5, 6]. With regard to skeletal regenerative potential, implantation of an ASC-seeded hydroxyapatite-coated poly (lactic-co-glycolic acid) scaffold into a critical-sized calvarial defect resulted in significant healing of the defect within 8 weeks [7, 8]. Finally, the adipogenic and angiogenic capabilities of ASCs have been utilized in the technique of cell-assisted lipotransfer (CAL), in which fat grafts are enriched with their native ASCs to improve retention and variability [9–12].

However, there exist a variety of different methods to obtain adipose tissue in clinical practice, with unclear impact on the viability and regenerative potential of ASCs. The current standard method for fat harvest for regenerative medicine purposes is liposuction. Specifically, suction-assisted lipoaspiration (SAL) [13], which uses manual movement of a small suction cannula to mechanically disrupt the adipose tissue, is most widely used [14, 15]. Previous work from our laboratory has demonstrated that relative to SAL, laser-assisted liposuction (LAL) leads to reduced ASC viability and in vivo regenerative potential [16], while ultrasound-assisted liposuction (UAL) does not affect ASC yield, proliferation, differentiation or capacity for tissue regeneration [17]. However, it remains to be determined what effects SAL itself has on key ASC characteristics. Therefore, the aim of this study was to determine the effects of SAL on ASC yield, viability, in vitro adipogenic and osteogenic differentiation capabilities, as well as in vivo regenerative potential by comparing ASCs derived from SAL lipoaspirates and those from resected adipose tissue.

## Methods

### Human adipose tissue collection and stromal vascular fraction isolation

Human adipose tissue was obtained from three healthy female donors after informed consent under approval of the Stanford University Institutional Review Board (Protocol no. 2188). Both abdominoplasty and suction-assisted lipoaspiration specimens were collected from each patient. Patients were female, 36–54 years of age, and had no known comorbidities. SAL was performed at a negative pressure of 760 mmHg using a 5 mm rounded, blunt cannula.

Lipoaspirate was processed to obtain the stromal vascular fraction as described previously [2]. Briefly, lipoaspirate was washed with sterile phosphate-buffered saline, followed by removal of the oil and blood/saline layers. The remaining fat layer was digested with Type II collagenase (Sigma-Aldrich; St. Louis, MO) in Medium 199 (Cellgro; Manassas, VA, USA) in a 37 °C water bath at 180 rpm for 30 min. The mixture was centrifuged at 1500*g* for 20 min at 4 °C and the supernatant was discarded. The cellular pellet was re-suspended in Dulbecco’s Modified Eagle’s Medium (DMEM) (Invitrogen; Carlsbad, CA, USA) with 10 % fetal bovine serum (FBS), filtered through a 100 µm pore size cell strainer (Corning; Corning, NY, USA), centrifuged at 300*g* for 15 min, and the supernatant was discarded once again. The cell pellet was then re-suspended in red cell lysis buffer and centrifuged once more time before re-suspending the stromal vascular fraction (SVF) in complete medium. Excised abdominoplasty specimens were de-epithelialized, mechanically minced into small pieces, and then digested and processed in the same manner as the lipoaspirate samples.

### Fluorescence-assisted cell sorting analysis

Our group recently demonstrated significant differences in the transcriptional profiles of primary ASCs when compared to cultured cells stressing the importance of using primary or very early passage cells in in all translational studies [18]. Therefore we utilized freshly isolated SVF and stained it for immediate fluorescence-activated cell sorting (FACS) to identify the ASC fraction. ASCs were defined by the established surface marker profile CD45-/CD31-/CD34+ [16, 19, 20]. Mouse anti-human monoclonal antibodies CD31-PE, CD45-PeCy7, and CD34-APC (BD Biosciences; San Jose, CA, USA) were used and propidium iodide staining was employed to exclude dead cells. Analysis was performed using a BD FACSAria machine (BD Biosciences).

### In vitro viability assay

Freshly extracted ASCs from SAL and excisional fat were seeded into a 96-well plate for determination of viability by MTT assay (Vybrant MTT Cell Proliferation Assay Kit, Invitrogen; Carlsbad, CA, USA).

### In vitro osteogenic differentiation

ASCs derived from SAL lipoaspirates and excised adipose tissue at passage two were cultured in osteogenic differentiation media (ODM), containing 10 % FBS, 1 % penicillin/streptomycin, 100 μg/mL ascorbic acid, and 10 mM β-glycerol 2-phosphate [21]. An alkaline phosphatase assay (Sigma-Aldrich) was performed after 7 days in culture with ODM, and mineralization was assessed using Alizarin Red staining at day 14. Alizarin Red staining was extracted with 20 % methanol and 10 % acetic acid in distilled water, and quantified using a spectrophotometer at 450 nm.

Total RNA was harvested immediately prior to beginning osteogenic stimulation with ODM at day 0, then again at day 7 and day 14 in osteogenic culture, and processed using the RNeasy Mini Kit (Qiagen; Hilden, Germany). Reverse transcription was performed using TaqMan Reverse Transcription Reagents (Invitrogen). An ABI Prism 7900HT Sequence Detection System (Applied Biosystems; Foster City, CA, USA) was used to perform quantitative real-time polymerase chain reaction (qRT-PCR) with Power SYBR Green PCR Master Mix (Applied Biosystems) as the reporter. qRT-PCR analysis was conducted to detect gene expression levels of the early osteogenic marker Runt-related transcription factor-2 (*RUNX*-*2*) as well as the late osteogenic marker osteocalcin (*OCN*). Expression levels of *RUNX*-*2* and *OCN* were normalized to beta-actin expression values.

### In vitro adipogenic differentiation

Cells from both groups were passaged twice and seeded in standard 6-well plates in triplicate at equal density. After reaching 70 % confluence, ASCs were cultured in adipogenic differentiation medium (ADM), consisting of DMEM, 10 % FBS, 1 % penicillin/streptomycin, 10 μg/mL insulin, 1 μM dexamethasone, 0.5 mM methylxanthine, and 200 μM indomethacin. Lipid accumulation was determined using Oil Red O (ORO) staining after 7 days in culture with ADM. Staining was imaged using a Leica DC300 camera and Leica DM IL inverted contrasting microscope at 10× magnification, then extracted with isopropanol, and quantified by absorbance spectrophotometry at 520 nm.

Total RNA was harvested at day 0 and day 7 of adipogenic induction culture. Expression levels of the adipogenic differentiation markers peroxisome proliferator-activated receptor γ (*PPAR*-*γ*), fatty acid binding protein 4 (*FABP4/AP2*), and lipoprotein lipase (*LPL*) were determined at two time points during adipogenic differentiation. Gene expression values were normalized to beta-actin.

### Animals

All mice were housed in the Stanford University Veterinary Service Center in accordance with NIH and institution-approved animal care guidelines. All procedures were approved by the Stanford Administrative Panel on Laboratory Animal Care.

### In vivo excisional wound model

Nude male Crl:CD-1-*Foxn1*^nu^ mice (Charles River Laboratories, Wilmington, MA, USA http://www.criver.com) between 8 and 12 weeks of age were randomized to three treatment groups: unseeded hydrogel control or hydrogel seeded with human ASC isolated from SAL lipoaspirates or resected adipose tissue. Pullulan-collagen hydrogel was produced as and seeded as described previously [5, 22]. Briefly, 2.5 × 10^5^ human ASCs suspended in 15 μL of PBS solution were pipetted onto hydrophobic wax paper and the hydrogel absorbed the cells actively by capillary, hydrophobic and entropic forces [5]. As previously described [23], two 6 mm full thickness wounds were created at the dorsum of each mouse. Each wound was held open by donut shaped silicone rings sutured on with 6-0 nylon sutures to prevent wound contraction and allow for healing by granulation. Wounds were covered with an occlusive dressing (Tegaderm, 3 M, St. Paul, MN, http://www.3m.com). Photographs were taken on days 0, 3, 5, 7, 9, 11, 13 and 15 and wound area was measured using ImageJ software (National Institute of Health, Bethesda, MD, http://www.nih.gov) (n = 8 wounds/group).

### Assessment of wound vascularity

To evaluate wound vascularity, immunohistochemical staining for the endothelial cell marker CD31 was performed as described previously (n = 8 wounds/condition) [22]. Briefly, wounds were harvested upon closure and processed for paraffin sectioning. Seven micron thick paraffin sections were stained with primary antibody (1:100 Rb α CD31, Ab28364, Abcam, Cambridge, UK, http://www.abcam.com) overnight at 4 °C, followed by secondary antibody staining (1:400 AF547 Gt α Rb, Life Technologies, Grand Island, NY, USA http://www.lifetechnologies.com). Cell nuclei were visualized with the nuclear stain DAPI. ImageJ (National Institute of Health, Bethesda, MD, USA http://www.nih.gov) was used to binarize immunofluorescent images taken with the same gain, exposure, and excitation settings as previously described [22]. Intensity threshold values were set automatically and quantification of CD31 staining was determined by pixel-positive area per high power field.

### Statistical analysis

Data are shown as mean ± SEM. Statistical analyses were performed using GraphPad Prism software (GraphPad Software, Inc.). Statistical comparisons were made using Student’s t-tests and ANOVAs, with Bonferroni corrections for multiple comparisons where appropriate. A *p value of < 0.05 was considered statistically significant.

## Results

### SAL yields a decreased frequency of ASCs

**Fig. 1:**
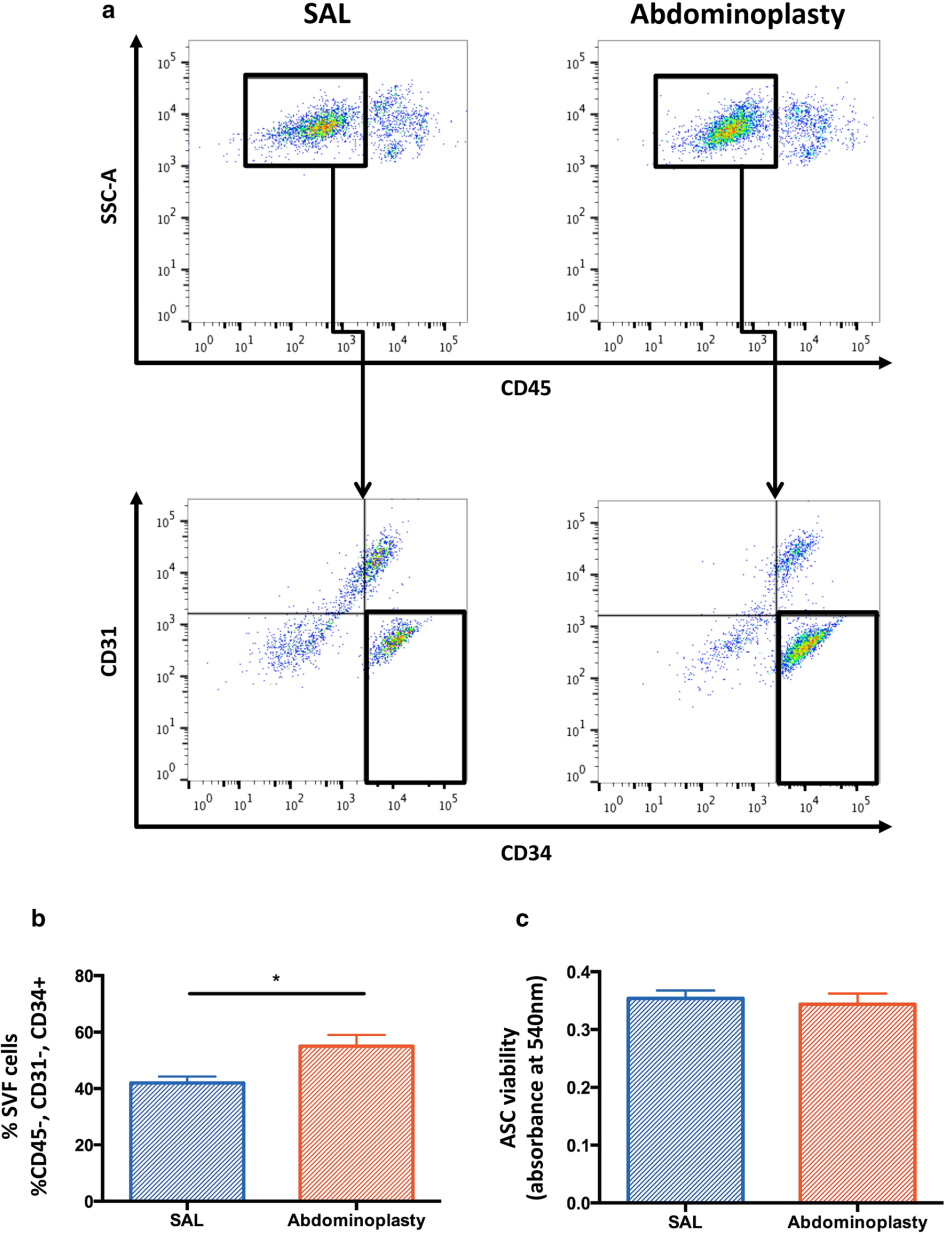
ASCs are less frequent in SAL lipoaspirates than in resected adipose tissue but display comparable viability. **a** Flow cytometric analysis evaluating the frequency of CD45- cells (*top row*) and ASCs (CD45-/31-/34+ cells; *bottom row*) within the SVF from SAL lipoaspirates and excised adipose tissue. **b** Quantification of CD45-/31-/34+ ASCs in SAL and excised adipose tissue derived. **c** MTT assay demonstrating no significant difference regarding cellular viability. n = 3. All data are means ± one SEM. *SAL* suction-assisted liposuction

ASC yield was assessed in freshly harvested SVF from lipoaspirates and resected adipose tissue to determine if SAL impacts ASC frequency. FACS analysis showed greater frequency of ASCs in SVF harvested from excisional fat compared to those from SAL (*p < 0.05) (Fig. 1a). The average yield of ASCs in processed SAL specimens was 42.4 %, and in processed abdominoplasty specimens was 55.8 % (Fig. 1b).

### SAL does not compromise ASC viability

A reduction of ASC yield in SAL lipoaspirates, did not result in an impaired ASC viability. An MTT assay was performed to assess the impact of SAL on ASC viability when compared to excision. Cell viability was not significantly different between ASCs harvested by SAL or excision (*p* = 0.53) (Fig. 1c).

### Osteogenic differentiation potential

**Fig. 2:**
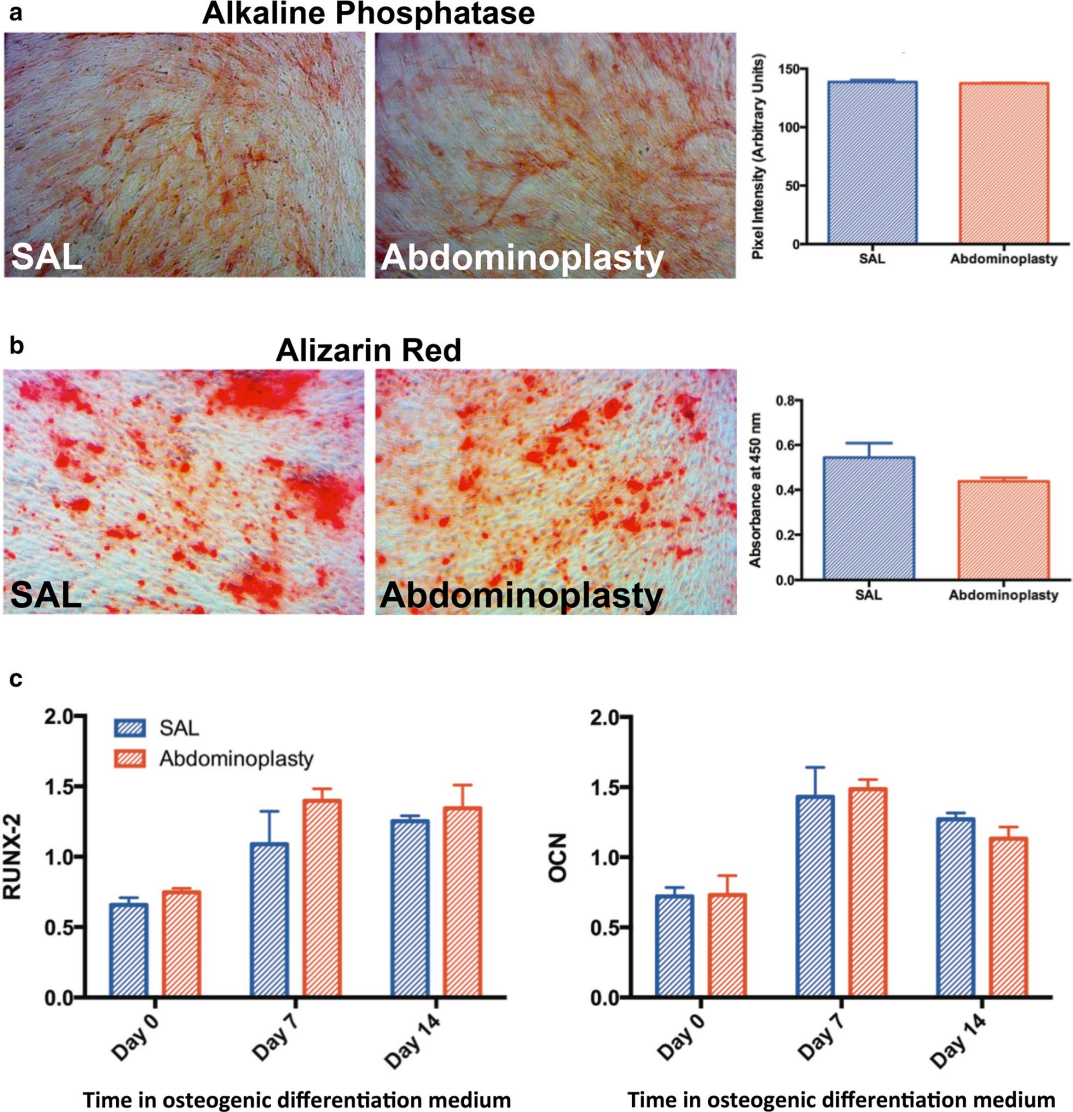
SAL and excisional fat derived ASCs have equal osteogenic lineage differentiation capacities. **a** Representative images and quantification of Alkaline Phosphatase and **b** Alizarin Red staining following osteogenic differentiation of SAL and excisional fat derived ASCs. **c** RT-PCR quantifying the expression of early (*RUNX2*), and late (*OCN*) osteogenic markers in vitro. n = 3. All data are means ± one SEM. *RUNX2* runt-related transcription factor 2, *OCN* osteocalcin

In order to determine the osteogenic differentiation potential of ASCs isolated from either SAL or excisional fat samples, ASCs were cultured in ODM for 14 days. There were no significant differences detected in alkaline phosphatase activity after 7 days in ODM (*p* = 0.44) (Fig. 2a) or in mineralization of the extracellular matrix at day 14, as measured by Alizarin Red assay (*p* = 0.06) (Fig. 2b). Similarly, there were no significant differences in expression of the osteogenic differentiation markers *RUNX*-*2* or *OCN* between the SAL- and excision-derived ASCs, at any time points assessed (Fig. 2c).

### Adipogenic differentiation potential

**Fig. 3:**
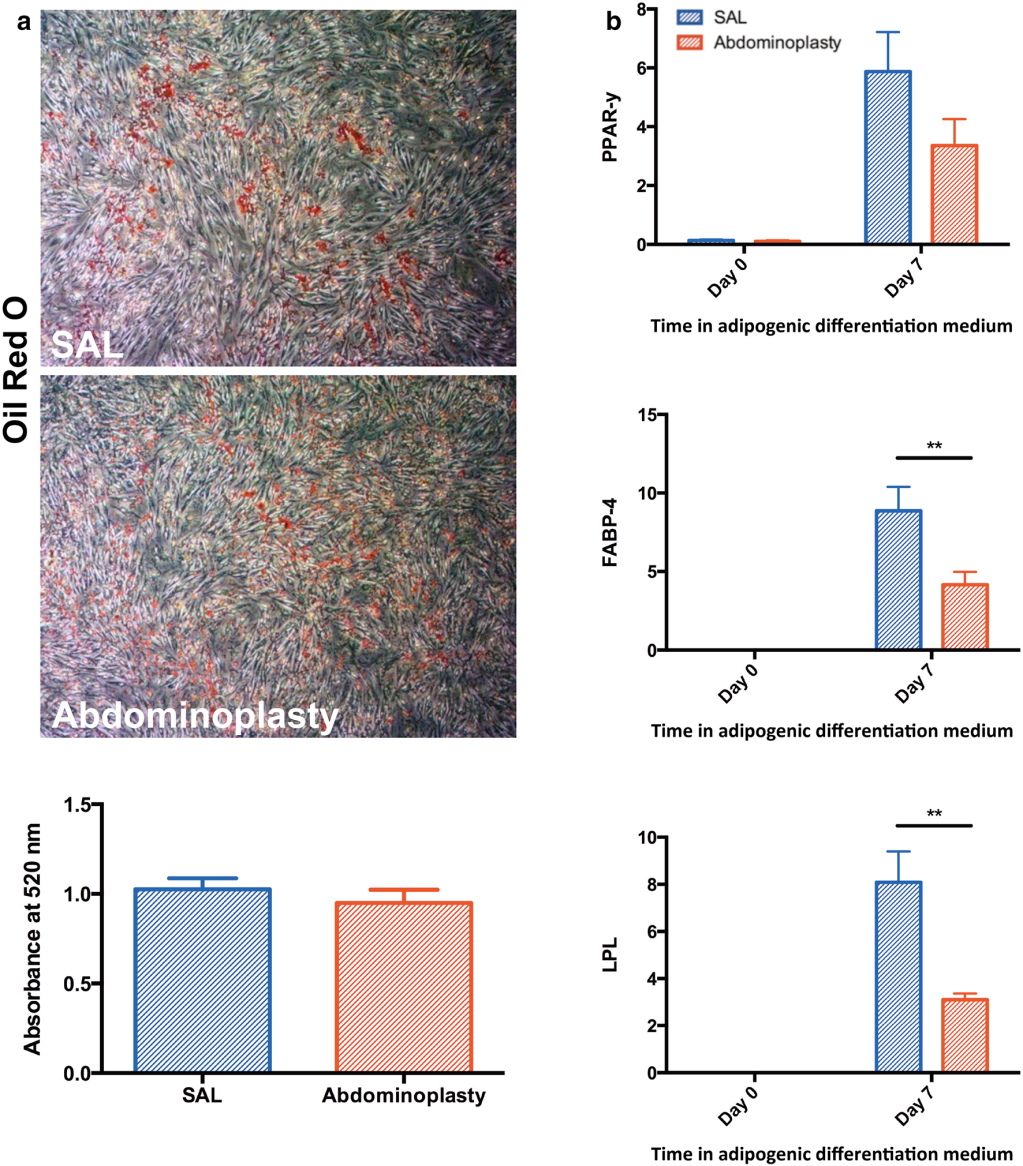
SAL derived ASCs have similar adipogenic lineage differentiation capacities. **a** Representative images and quantification of Oil Red O staining following adipogenic differentiation of SAL and abdominoplasty derived ASCs. **b** RT-PCR quantifying the expression of adipogenic markers in vitro. *Top* PPAR-γ, *middle* FABP4, *bottom* LPL. n = 3. All data are means ± one SEM. *PPAR-γ* peroxisome proliferator-activated receptor γ, *FABP4* fatty acid binding protein 4, *LPL* lipoprotein lipase

Cells were cultured in ADM for 7 days to determine adipogenic differentiation potential of ASCs derived from SAL or excisional fat. Lipid accumulation was confirmed by ORO staining after 7 days of culture in ADM, indicating appropriate adipogenic differentiation of ASCs. Quantification of Oil Red O staining showed no significant differences between the two groups (*p* = 0.47) (Fig. 3a). Additionally, RNA was harvested prior to induction of differentiation at day 0 and again after 7 days of culture in ADM to correlate adipogenic marker transcript expression levels with observed in vitro adipogenic differentiation. Interestingly, gene expression of the intermediate and late adipogenic differentiation markers *FABP*-*4/AP2* and *LPL* were significantly enhanced in ASCs harvested by SAL compared to those from excisional fat tissue (**p < 0.01) (Fig. 3b). This difference was seen after 7 days of adipogenic induction, but not at day 0 before induction of differentiation in ADM. Similarly, gene expression of the early adipogenic marker *PPAR*-*γ* showed a trend toward greater expression at day 7 in cells derived from SAL, however this did not reach statistical significance (*p* = 0.06).

### SAL and excisional fat derived ASCs equally enhance wound healing

**Fig. 4:**
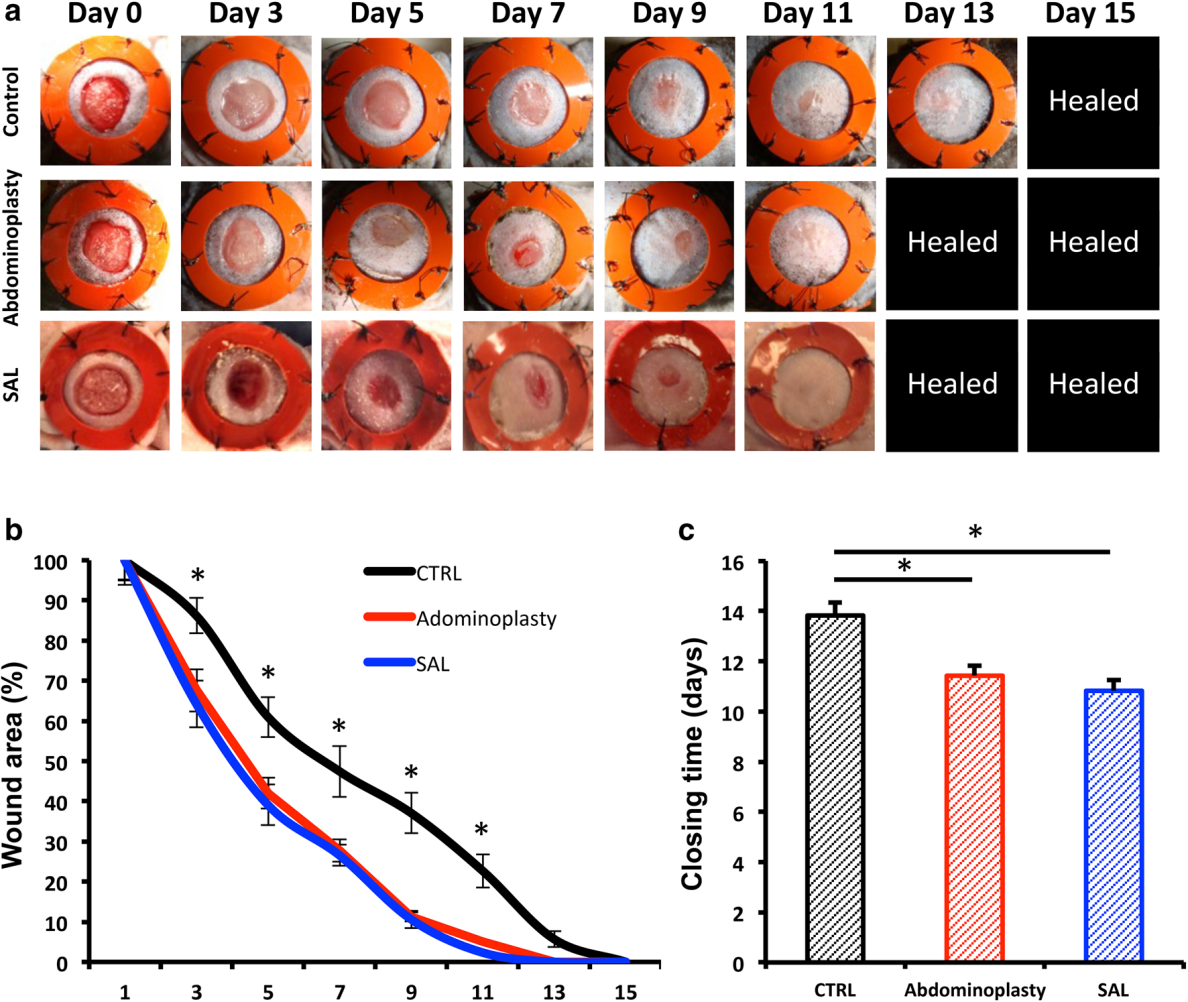
Application of SAL and abdominoplasty derived ASCs are equalliy efficacious to enhance cutaneous healing. **a** Gross appearance, **b** wound healing kinetics, and **c** closing times of humanized excisional murine wounds treated with hydrogel seeded hASCs harvested via SAL, abdominoplasty or unseeded hydrogel. n = 8. *Asterisk* indicates *p* ≤ 0.05. All data are means ± one SEM

To evaluate the therapeutic functionality of SAL derived ASCs versus ASCs isolated from excisional fat in vivo, cell-seeded hydrogels [5] were applied to a previously established model of murine cutaneous healing [5, 24]. Consistent with their unimpaired in vitro functionality, ACSs obtained via SAL demonstrated comparable therapeutic efficacy for cutaneous regeneration versus cells isolated from abdominoplasty samples (Fig. 4a). Both ASC treatment groups displayed significantly improved healing kinetics as early as day three compared to unseeded hydrogel controls (Fig. 4b). The accelerated healing rates directly resulted in significantly faster wound closure times in the ASCs groups (11.4 and 10.8 vs. 13.8 days, **p < 0.01) (Fig. 4c). These data indicate that ASCs derived from either SAL or excision both have a positive effect on in vivo regeneration.

### SAL and excisional fat derived ASCs both enhance wound vascularity

**Fig. 5:**
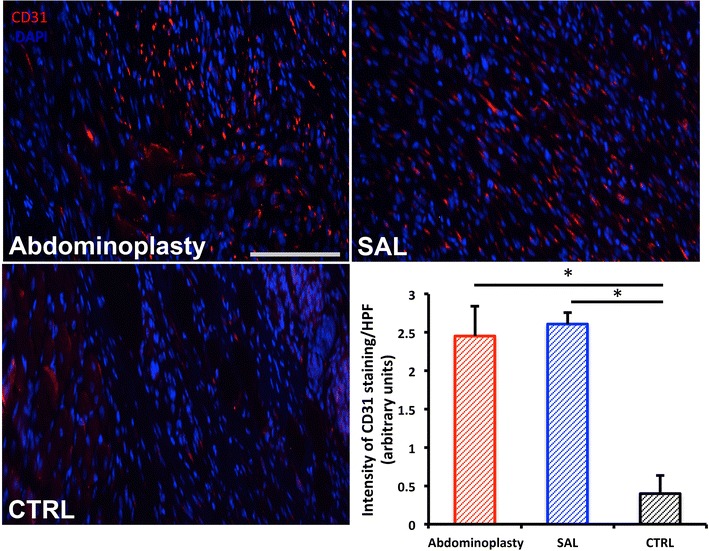
Both ASC treatment groups display enhanced cutaneous wound vascularity. CD31 staining confirmed a significant increase in neovascularization among both ASC tretment groups. DAPI nuclear stain. *Scale bar* 100 μm. n = 8. *Asterisk* indicates *p* ≤ 0.05. All data are means ± one SEM

Improvement of wound healing by ASCs is widely attributed to enhanced vascularization of the wound bed [17, 25–28]. Indeed, both ASC treatment groups showed significantly enhanced neovascularization compared with acellular scaffold controls (*p < 0.05), confirming our results regarding in vivo regenerative potential (Fig. 5). Similar to the wound healing outcomes, no significant differences between the two ASC groups could be detected. This further corroborates that in vivo regenerative potential is preserved in SAL-derived ASCs.

## Discussion

Despite exciting discoveries regarding the regenerative potential of ASCs, the use of ASCs in clinical practice is still in its infancy. This is in part due to an incomplete understanding of how various harvesting and processing methods affect ASC biology. Several different methods for fat harvest exist, whether for fat grafting in the operating room or for the isolation of ASCs in the laboratory [29–32]. With the goal of decreasing donor site morbidity with increased operative efficiency, new harvesting techniques have been developed beyond the traditional technique of resection, such as SAL, UAL, LAL, and power-assisted liposuction (PAL). However, while some of these methods may offer improvements in clinical variables such as decreased operative time, improved skin retraction, and minimized blood loss [33], the current body of literature regarding the comparative effects of these methods on preservation of the cellular contents is largely incomplete and often inconsistent. These potential effects are of interest to translational researchers utilizing ASCs in regenerative therapies, as well as to clinicians looking to improve viability of transferred fat grafts. To our knowledge, there has been no study to date that determines the effects of SAL on proliferation, differentiation potential, and wound healing when compared to the standard of excisional fat. We evaluate the characteristics of SAL and resection-derived ASCs in a laboratory setting with paired samples from patients who serve as their own controls. These controlled conditions enable an exact assessment potentially superior to clinical comparisons utilizing one-step cell isolation protocols in the operating room.

In this study, we found that ASCs obtained from both SAL and excised abdominoplasty tissue occurred at high frequencies and viability, although excised adipose tissue provided greater yields of ASCs when compared to SAL. Factors influencing ASC yield have been discussed controversially in the literature. In addition to harvesting technique, patient demographics can affect ASC frequency in adipose tissue. Generally, there are no detectable differences in ASC yield or proliferation with age [19, 34]. However, high donor age and comorbidities such as diabetes significantly impair ASC functionality [19, 35] and donor gender affects ASC properties, with more robust osteogenic differentiation in ASCs from male patients [36]. Furthermore, previous studies from our group have demonstrated depot-specific differences in ASCs, with ASCs isolated from the flank and thigh showing greater osteogenic potential but ASCs from the flank having lesser adipogenic capabilities when compared to the arm and abdomen [37].

The capability of ASCs to differentiate down multiple lineages is of critical importance in their utility in tissue engineering and cell-based regenerative therapies. Previous studies from our group have found no significant difference in osteogenic differentiation potential between suction-assisted lipoaspiration and third-generation ultrasound-generation lipoaspiration, despite the mechanical disruption delivered during ultrasound application [17]. In contrast, ASCs derived from LAL have been shown to suffer from decreased osteogenic differentiation capacity relative to those from SAL [16]. The results from this study demonstrate that SAL does not impair the osteogenic differentiation potential of ASCs. This is not surprising, since SAL delivers a mechanical effect rather than heat, and is thus an approach more similar to UAL than LAL.

Interestingly, we found that expression of adipogenic differentiation markers FABP-4 and LPL were significantly higher in SAL derived ASCs when compared to those harvested from excisional abdominoplasty fat tissue. Expression of the early adipogenic marker PPAR-γ was also greater, although not significantly so. Our findings corroborate those in a recent study by Keck et al. [38], who determined expression of adiponectin, PPAR-γ, and GLUT4 to be significantly increased in ASCs from PAL, a technique analogous to SAL, when compared to manual aspiration. A potential explanation for these findings may be found in the cellular effects of mechanotransduction, the conversion of mechanical forces to biochemical signals [39]. It is becoming increasingly probable that ASCs are subject to significant mechanotransductive effects [40, 41], much as are other progenitor cell types. However, the literature is still developing in this area and results thus far have been inconsistent and largely focused on either adipocytes or adipose tissue as a whole [42, 43]. Due to the conflicting data regarding mechanotransduction in ASCs, one may turn to the BM-MSC literature for potential clues. Importantly, shear stress has been previously shown to cause changes in cytoskeletal distribution in MSCs, ultimately leading to alterations in differentiation potential. Specifically, Chang et al. [44] found that shear stress led to increased expression of the early adipokine PPAR-γ and decreased expression of the early osteogenic gene RUNX-2 [44]. Here, we see similar effects in ASCs, with trends toward increased PPAR-γ and decreased RUNX-2 expression in ASCs isolated from lipoaspirates when compared to those obtained from excised adipose tissue. Therefore we may conclude that mechanical forces exerted during lipoaspiration alters ASC biology, at least at the transcriptional level. However, further work is needed to clarify these potential effects.

ASCs harbor great promise for tissue regeneration applications [5]. In this study we demonstrate that SAL-harvested ASCs have an identical potential for the enhancement of cutaneous healing when compared to ASCs derived from excisional fat. Furthermore, wounds treated with either ASC population displayed significantly greater vascularity compared to an unseeded scaffold control group. These promising findings distinguish SAL as a reliable method for obtaining ASCs suitable and effective for regenerative medicine approaches, and an equivalent source of ASCs when compared to those derived from three-dimensionally intact adipose tissue.

## Conclusion

ASCs represent a promising source of multipotent cells for tissue engineering and regenerative medicine. Suction-assisted lipoaspiration offers a possibility for relative ease of harvest of ASCs with minimal donor site morbidity. Here we show that SAL lipoaspirates provide a slightly decreased yield of viable ASCs when compared to resected adipose tissue. ASCs derived from SAL retain full multipotency and regenerative capabilities. Overall, these findings suggest that SAL is a reliable and effective method of obtaining ASCs for tissue engineering approaches and cell-based therapies when compared to the gold standard of minimally-manipulated excisional adipose tissue, and does not damage ASCs in terms of viability, osteogenic and adipogenic differentiation capacity, wound regenerative potential, or wound neovascularization.
